# Novel Formulation of Eye Drops Containing Choline Salicylate and Hyaluronic Acid: Stability, Permeability, and Cytotoxicity Studies Using Alternative Ex Vivo and In Vitro Models

**DOI:** 10.3390/ph14090849

**Published:** 2021-08-26

**Authors:** Katarzyna Barbara Wróblewska, Bartłomiej Milanowski, Małgorzata Kucińska, Szymon Plewa, Jolanta Długaszewska, Izabela Muszalska-Kolos

**Affiliations:** 1 Department of Pharmaceutical Technology, Poznan University of Medical Sciences, Grunwaldzka 6, 60-780 Poznan, Poland; bmilan@ump.edu.pl; 2 GENERICA Pharmaceutical Lab, Regionalne Centrum Zdrowia Sp. z o.o., Na Kępie 3, 64-360 Zbąszyń, Poland; 3 Department of Toxicology, Poznan University of Medical Sciences, Dojazd 30, 60-631 Poznan, Poland; 4 Department of Inorganic and Analytical Chemistry, Poznan University of Medical Sciences, Grunwaldzka 6, 60-780 Poznan, Poland; splewa@ump.edu.pl; 5 Department of Genetics and Pharmaceutical Microbiology, Poznan University of Medical Sciences, Święcickiego 6, 60-781 Poznan, Poland; jdlugasz@ump.edu.pl; 6 Department of Pharmaceutical Chemistry, Poznan University of Medical Sciences, Grunwaldzka 6, 60-780 Poznan, Poland; imuszals@ump.edu.pl

**Keywords:** choline salicylate, hyaluronic acid, eye drops, SIRC cell line, alternative models

## Abstract

This work investigated the potential of a novel formulation of eye drops containing a non-steroidal anti-inflammatory drug—choline salicylate (CS)—and hyaluronic acid (HA). Thus, these drops may exert both anti-inflammatory and regenerative activity. The experiment was conducted through the careful characterization of physicochemical properties, stability, and quality of eye drops. Moreover, microbiological analysis, as well as penetration and cytotoxic studies, were performed. The UV, HPLC-UV, and HPLC-MS/MS methods were used to determine the purity and stability of CS. The penetration rate of CS was assessed using a hydrophilic membrane and ex vivo porcine cornea model. Additionally, the cytotoxic effects were evaluated using the SIRC cell line. The interaction between HA and CS was tested using size-exclusion chromatography and IR spectrophotometry. As a result, HA increased the viscosity of the drops, which prolonged their contact with the ocular surface, thus ensuring more effective penetration of CS into the corneal structure. After long-term storage, an interaction in the pharmaceutical phase between CS and HA was observed. However, this interaction did not affect the viability of rabbit corneal cells. Our findings showed that eye drops with CS and HA, stored at 2–8 °C in light-protected conditions, met the criteria of stability and safety.

## 1. Introduction

Local drug administration is still the main route of ocular drug application to treat many eye diseases; e.g., dry eye syndrome and conjunctivitis. The conventional treatment given is a course of chosen eye drops. However, one notable disadvantage of this form of the drug is the short retention period on the surface of the eye. After application, there is a rapid removal of exogenous substances under the influence of defense mechanisms, such as blinking or tearing. Achieving active pharmaceutical ingredient (API) concentrations at the pharmacological level would require more frequent application, which increases the risk of local and systemic side effects [1].

The use of polymers with a high molecular weight and viscosity, which can bind significant amounts of water, is one of the most common technological strategies for extending the contact time with the surface of the cornea and conjunctiva, thus increasing the bioavailability of the preparation [2]. In addition, these compounds soothe, moisturize, and protect the surface of the eye, reducing the symptoms of dry eye syndrome. For this purpose, mainly cellulose derivatives such as hydroxypropylmethylcellulose (HPMC), hydroxyethylcellulose (HEC), and methylcellulose (MC) are used today, as well as polyvinyl alcohol (PVA), carbomer, propylene glycol, and dextran. These polymers are well tolerated at the concentrations found in commercial eye drops. However, preparations with a higher viscosity may cause visual acuity disturbances after application and leave a dry film. Some of these polymers have Newtonian properties; i.e., they do not shear under the influence of blinking, causing them to become less spread out over the eye surface, and therefore less effective. Therefore, polymers characterized by non-Newtonian and pseudoplastic properties, such as hyaluronic acid (HA), are becoming increasingly popular [3].

**Figure 1:**
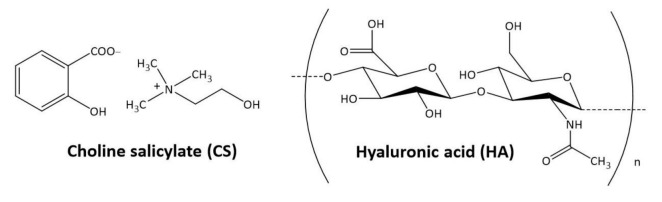
Structural formulas of choline salicylate (CS) and hyaluronic acid (HA).

Hyaluronan is a biopolymer with a linear structure. It belongs to the group of compounds called glycosaminoglycans (GAGs) [4]. The polymer’s molecule is made of D-glucuronic acid and N-acetylglucosamine (Figure 1), connected by alternating glycosidic bonds β (1–4) and β (1–3) [5]. However, hyaluronan differs from the others GAGs. It has a larger molecule (about 25,000 disaccharide units), and therefore a higher molecular weight of up to 10 MDa. Unlike other GAGs, hyaluronan is the backbone to which other glycosaminoglycans and proteoglycans attach through binding proteins to form huge aggregates.

HA is an essential component of the extracellular matrix (ECM) [4,6]. It should be emphasized that hyaluronic acid is a natural compound with the same chemical structure in all vertebrates and bacteria [7]. It is a highly biocompatible compound that does not exhibit cytotoxic effects and does not induce immunogenic reactions [4,7]. In living organisms, it is in the form of sodium salt. Hyaluronic acid can bind large amounts of water, which is possible through the covalent bonding structure and consequent negative charge that water possesses. This compound increases the osmolality of solutions and has unique viscoelastic properties [6]. Due to these properties, HA eye drops perfectly mimic the conditions on the eye surface. Tear fluid has a characteristic rheological profile: it is sticky under static conditions, but when blinking, its viscosity decreases. In HA solutions, the shear stress causes the HA molecules to line up parallel to each other during blinking. The solution temporarily loses its viscosity and easily spreads over the surface of the cornea. The HA chains form a tangled mesh between blinks, making the solution more viscous [8]. It also has mucoadhesive properties, thanks to which it can closely interact with the mucin layer, covering the surface of the cornea and conjunctiva. This stabilizes the precorneal tear film and maximizes the residence time of the solution on the eye surface, where HA can improve the hydration and lubrication of the eye due to its hygroscopic and adhesive properties [9]. In addition, it has been shown that 0.3% HA solution stimulates the migration of corneal epithelial cells and has anti-inflammatory and antioxidant properties, and therefore may consequently play a role in wound healing processes [10].

HA is a natural component of the human eye: it has been found in the vitreous body, lacrimal gland, corneal epithelium, and in tear and conjunctival fluid [11]. The vitreous concentration of HA is approximately 190–320 µg/mL, of which 45–77% is high-molecular-weight HA (>1000 kDa). Hyaluronic acid is also a component of the tear film [12]. It has become a widely used ophthalmic product due to its safety, and the defensive properties it possesses in protecting the corneal endothelium. HA plays an important role in ophthalmic surgery during anterior segment surgery [9]. Due to its viscoelastic, lubricating, cushioning, moisturizing, and other rheological properties, HA promotes tissue healing by increasing the proliferation and migration of corneal epithelial cells and readhesion of the retina. After ophthalmic surgery, HA stabilizes the tear film, shortens the healing time, minimizes the risk of adhesions, reduces the formation of free radicals, and normalizes intraocular pressure. HA solutions are the most commonly used fluids to protect and lubricate the delicate tissues of the eye, replenish lost vitreous fluid, and provide room for manipulation during ophthalmic procedures. HA’s viscosity keeps tissues in place, thereby reducing the risk of dislocation [9].

In addition, HA is the active ingredient in many eye drops, which are essential in the treatment of conditions such as dry eye syndrome by moisturizing the surface of the eye and improving vision quality. New hyaluronan derivatives with improved mechanical and biological properties are currently being investigated to develop eye drops with extended residence time in the eye. For example, promising preliminary results were obtained with HA–cysteine ethyl ester and urea-crosslinked HA (HA-CL) [13]. Crosslinking is a chemical strategy aimed at increasing the stiffness of the polymer network, extending its durability at the application site, reducing its susceptibility to enzymatic degradation, and thus reducing the frequency of application of the formulation [14]. Several studies showed that a polyanionic polysaccharide such as HA might improve the spreading of meibomian and tear lipid films on the eye surface [15,16]. Georgiev and colleagues showed that HA interacted with meibomian lipids, enhancing the tear film stability [17]. It was observed that in healthy volunteers, even a single application of HA increased the tear film thickness (TFT) for up to 20 min [18]. In addition, Szegedi et al. found that a single HA application via eye drops may increase TFT in patients with dry eye syndrome [19]. Moreover, in vivo studies confirmed the increase in the thickness of the tear film (from 60 to 75 nm) induced by 0.1% HA solution in patients with lipid deficiency as a consequence of dry eye syndrome [20].

It has been observed that the biological functions of HA depend on the size of the polymer particles. High-molecular-weight (HMW) HA has anti-inflammatory and immunosuppressive effects. It inhibits phagocytosis and protects tissues from damage and apoptosis [10]. Medium-sized polymers have anti-inflammatory and angiogenic properties. The smallest oligosaccharides (LMV) have an antiapoptotic effect [10]. They can induce the production of IL-8, including in the bronchial epithelium, and therefore may play an important role in lung infections [21]. All the described properties make HA well suited for use in the form of artificial tears. Extensive research has been conducted to evaluate the safety and efficacy of hyaluronic acid eye drop solutions, with all studies confirming the effectiveness in relief of the symptoms of dry eye syndrome, with HA drops correlating with the concentration of HA and molecular weight (generally 0.1–0.4% solutions, 0.8–1.4 MDa HA) [8].

Acetylsalicylic acid is a powerful drug with known anti-inflammatory activity [22] that is also used, particularly as a combined therapy, in ocular surface inflammatory disease [23], glaucoma treatment [24], and acute central serous chorioretinopathy [25]. Thus, there is increased interest in the possibility of using choline salicylate, which is more advantageous in terms of physicochemical properties than acetylsalicylic acid, as an active ingredient of ocular anti-inflammatory drops [26]. As a nonacetylated derivative of salicylic acid, choline salicylate is classified as a first-generation nonsteroidal anti-inflammatory drug. When applied topically, it has a disinfecting effect and a weak bactericidal effect against some microorganisms, which, together with the analysis of its physicochemical properties, allows the composition of an ophthalmic drug to be developed in the form of 2% eye drops with increased viscosity. The studies conducted on the penetration of CS through hydrophilic membranes and the porcine cornea have confirmed the possibility of its penetration into the corneal structures, justifying the research on the development of an ophthalmic drug. Our previous study showed that the combination of CS and HPMC in eye drops met the quality criteria: pH, viscosity, and osmolarity, as well as in terms of microbiological purity [26]. HA has several advantages, including mucoadhesive, moisturizing, lubricating, antioxidizing, and anti-inflammatory properties [9,10], which are important for ophthalmological drugs. Thus, it was decided to evaluate the possibility of combining two active ingredients, choline salicylate and hyaluronic acid (Figure 1), in eye drops intended to treat corneal diseases.

Earlier studies on the use of CS eye drops [27], and the results of the stability of CS and HPMC eye drops [26], justify the need for further research to improve formulation and the technology of eye drops combining choline salicylate with hyaluronic acid. For this purpose, the technology of preparing sterile eye drops containing CS (2%) and HA (0.4%) as a viscosity-enhancing agent was developed. The microbiological purity, cytotoxicity, penetration through an artificial hydrophilic membrane, and the porcine cornea were investigated for this new formulation. Stability studies were performed under controlled conditions (accelerated and long-term tests), under uncontrolled conditions (2–8 °C), and during exposure to light (photodegradation). Bearing in mind the literature reports stating that HA is an unstable molecule, at a temperature of 100 °C and in solution, HA chains are degraded, which results in a reduction of the molecular weight and viscosity [28,29,30,31]. The study of the influence of the sterilization parameters with saturated steam under pressure (temperature and time) on the viscosity and cytotoxicity of 0.4% HA solutions was performed. The research used a method based on HPLC-UV, HPLC-MS/MS chromatography and UV, and IR spectrophotometry.

## 2. Results and Discussion

### 2.1. Choline Salicylate Permeation through Membranes

The penetration rate of CS from the eye drops and the CS solution in isotonic sodium chloride solution was assessed using a hydrophilic membrane made of regenerated cellulose (Visking^®^) and a porcine cornea. The CS content in the acceptor part of the Franz cell (volume 8 mL, penetration area 1 cm^2^) was determined by the UV method [26]. The blank test confirmed that the solutions of the acceptor part did not show UV absorption in the region of interest (blank sample absorbance was ˂0.004).

**Figure 2:**
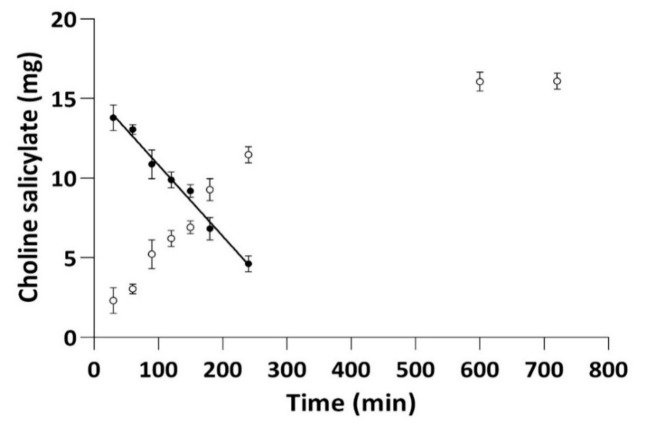
The ability of CS to penetrate the hydrophilic membrane (regenerated cellulose) for eye drops with CS (2%) and HA (0.4%). Plots of the relationship: the content of CS in the acceptor part of the Franz cell as a function of time; *Q* = f(t) (○) and differential chart *Q*_∞_ − *Q_t_* = f(t) (●).

To investigate the kinetics of CS permeation through membranes, the following kinetic models were applied: the zero-order model (used for pharmaceutical dosage systems that do not disintegrate and have a very slow drug release) [32]; the first-order model (used to describe the absorption and release of water-soluble drugs from porous matrices); the Higuchi model (used to describe the release of soluble and sparingly soluble drugs in aqueous media, from various semisolid and/or solid matrices); and the Korsmeyer–Peppas model, which is a generalized model of the Higuchi equation that allows one to explain drug-delivery mechanisms in which erosion and/or dissolution of the matrix occurs [32] (Table 1). The Korsmeyer–Peppas model is widely used to describe the drug release from polymer systems [32]. The concentration of CS in the acceptor part of the Franz cell changed as a function of time until a plateau was reached at 600 min from the experiment start (Figure 2). The results of the kinetic evaluation (Table 1) confirmed that the penetration of CS across the hydrophilic membrane for the tested drops showed the best fit to the zero-order model.

By comparing our results with those previously published [26], we showed that the process of CS penetration through a hydrophilic membrane from the isotonic environment was faster (52.3 µg·min^−1^) [26] than from eye drops with hyaluronic acid (45.0 µg·min^−1^) (Table 1). The addition of hyaluronic acid or HPMC slowed the CS penetration process through the hydrophilic membrane, and in both cases, this effect was comparable [26].

The simultaneous determination of CS in the acceptor and donor parts of the Franz cell (1 mL) (Table 2) confirmed that the CS did not pass through the cornea, but penetrated its structures. After 5 min and 3 h of exposure, there was an approximately 21.4% and 23.7% loss of CS content, respectively, from the donor part of the chamber filled with isotonic CS solution [26]. In the case of exposure of the cornea to CS drops with HA, the decrease was approximately 15.2% and 18.3% after 5 min and 3 h, respectively. Therefore, the level of CS in the cornea was assessed using the methodology described earlier [26], based on the determination of CS by the UV method after extraction from the cornea with a water–methanol mixture (1:2, *v*/*v*). Thus, from the isotonic solution to the cornea, approximately 6.6 ± 0.8% and 17.1 ± 1.1% of the CS penetrated after 5 min and 3 h exposure, respectively [26]. On the other hand, for eye drops with hyaluronic acid, the amount was approximately 7.8 ± 0.2% and 18.3 ± 0.2%, respectively (Table 2). Thus, similarly to HPMC, hyaluronic acid increased the viscosity of the drops, prolonged their contact with the eyeball, and contributed to more-effective penetration of CS into the corneal structure, which allowed for approximately 7.8% of the administered dose.

It is widely known that the availability of API by ocular tissue is limited by several mechanisms such as lachrymal drainage, systemic absorption, the small size of the ocular cavity, and existing anatomical barriers (mainly the corneal epithelium) [33,34]. Thus, the crucial parameter in ophthalmology is permeability to the anterior (composed of the aqueous humor, conjunctiva, cornea, iris, ciliary body, and lens) and posterior (composed of the choroid, optic nerve, retina, sclera, choroid, and vitreous humor) segments of the eye [33,35]. The literature data indicated that the most important paths by which the drug can enter the eye are the corneal and conjunctival routes [33,35]. In general, the conjunctiva is permeable to hydrophilic and large molecules, while most drugs used in clinics are relatively small and lipophilic structures [33]. Moreover, for the drugs applied topically, their bioavailability could be decreased mainly by lower cornea permeability and the washing-out effect by lacrimal fluid [36]. This makes cornea permeability testing one of the most important and widely studied aspects in research dedicated to ocular drug delivery. It should be highlighted that the cornea is less permeable than other important ocular tissue—the sclera [37], which is the limiting factor of drugs intended to reach the retina in terms of drug delivery. The rapid flow of blood from the choroid in the sclera layer may affect drug availability to the retina [37]. Thus, in the presented study, we only focused on cornea permeability. This also resulted from the fact that the tested formulation was dedicated to treating mainly corneal diseases.

### 2.2. Stability of Eye Drops

As part of assessing the stability of the prepared drops, the impact of storage conditions was tested: photodegradation, 40 °C/75% RH—accelerated test, 25 °C/60% RH—long-term test, and 2–8 °C—storage in a refrigerator under uncontrolled conditions. Single-dose minims (Eprus^®^ sterile minims eye drops, 1 mL) were proposed as the immediate packaging of the eye drops, the suitability of which was confirmed by a microbiological test.

**Figure 3:**
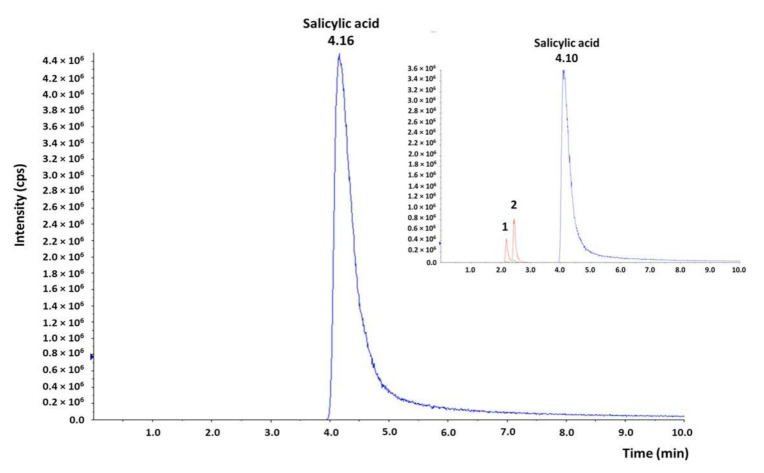
HPLC-MS/MS chromatograms of eye drops with CS (2%) and HA (0.4%) in immediate packaging (minims) after 3 months of storage at 40 °C/75% RH. The inset shows the HPLC-MS/MS chromatogram of eye drops in minims after exposure to light (250 W/m^2^; 1.2 × 10^6^ lux·h): 1—2,5-dihydroxybenzoic acid; 2—2,3-dihydroxybenzoic acid.

The solutions of the CS eye drops with HA, when exposed to light (250 W/m^2^; 1.2 × 10^6^ lux·h), changed color with a simultaneous loss of choline salicylate content at a level of 6.3% (HPLC-UV). The presence of the photodegradation products of CS, such as 2,3- and 2,5-dihydroxybenzoic acid, was observed in HPLC-MS/MS chromatograms (Figure 3). CS eye drops should be protected from light exposure by placing unit packages (minims) in collective packaging; e.g., a cardboard box.

For these studies, two different protocols were used to obtain the tested eye drops. In the first method (Preparation 1), HA and CS solutions were mixed, transferred to glass bottles (20 mL), sealed, and autoclaved at 121 ± 2 °C for 20 min. In the second procedure (Preparation II), CS solution was filtered through a sterilization filter (0.22 µm) into a sterile bottle and mixed with sterile HA solution. The bottle was closed with a sterile stopper under sterile conditions. The eye drops (Preparations I and II) were then aseptically transferred to the immediate packaging (Eprus^®^, 1 mL). Eye drops made according to Preparation II were used only in the stability studies under the accelerated and long-term test conditions, which resulted from observing the color changes of the drops made according to Preparation I. All other tests (permeation through membranes and porcine cornea, photostability, microbiological test, chromatographic purity, cytotoxicity, and size-exclusion chromatography) refer to the drops made according to Preparation I.

Glass bottles (20 mL) and minima (1 mL) were used as immediate packaging in the eye drop stability studies. Changes in CS content (HPLC-UV method), pH, viscosity, and osmotic pressure were observed. At the same time, a color change (browning) of the solutions was observed after 1 month of storage in minims (Preparation II), as well as after 6 months of storage in a bottle (Preparation I). Over time, this process intensified, which prompted Preparation II. After 3 months of storage, the declared content of CS in the minims increased to approximately 110% and 115%, respectively, for drops prepared using Preparations I and II. This was due to the leakage of the packaging under the conditions of the described test. This leak also affected the other parameters (Table 3). The drops stored in bottles for 6 months retained the appropriate quality parameters, keeping the CS content at the level of no more than 3.9% of the loss and complying with the initial value in terms of pH and osmolarity (Table 3). The optimal pH of eye drops is equal to the pH of the tear fluid, around 7.4. The Polish Pharmacopoeia (FP XII) allows pH values for eye drops from 3.5 to 8.5 and osmolarity of 280–320 mOsmol/L [38], but the *European Pharmacopeia* 10th Edition does not precisely specify the permissible pH values. The lowering of viscosity of the drops was observed during their storage at 40 °C. For Preparation I, the decrease after 6 months of storage in bottles was 3.4%, and 22.1% after 3 months of storage in minims. The viscosity of the drops prepared using Preparation II changed significantly after 6 months of their storage at 40 °C; i.e., 30.4% for bottles and 35.4% for minims (Table 3). When comparing the parameters of drops made using Preparations I and II, Preparation I was considered as appropriate. The drops prepared using this method were used for the cytotoxicity tests and interaction analysis.

A sterility test was used to evaluate the minims type packaging material. The studies were performed using two techniques (direct culture, membrane filters) with two media (TSB, TG). Under the study conditions, the developed eye drops showed no antimicrobial activity, which confirmed the usefulness of the methods used to assess microbiological purity. In sterility tests performed in the initial period and after 3 months of storage (40 °C/75% RH), no growth of microorganisms was observed either at the beginning. Bacterial growth was observed only in one case (Table 4). Thus, despite the leakage observed in the above-described test, the minims used (Eprus^®^ sterile minims 1 mL eye drops) met the sterility criteria. They do not guarantee tightness at elevated storage temperatures. Their use should not be excluded when stored at lower temperatures (25 °C or 2–8 °C).

Under long-term test conditions (25 °C/60% RH) and uncontrolled conditions in a refrigerator (2–8 °C), the stability of drops prepared using Preparation I stored for 12 months both in the bottle and in the minims was confirmed (Table 5). In bottles, the observed changes in CS content did not exceed 2%, while in the minima, they amounted to 3.5% at 25 °C and 4% in a refrigerator, respectively. Changes in the CS concentration in the drops prepared using Preparation II after 6 months of storage at 25 °C reached a value of 4.5%, and in minims after 6 months—3.5%. The osmolarity and pH of the tested solutions under these conditions did not change significantly (Table 5). In addition, during their storage at 25 °C, the droplet viscosity decreased. In drops prepared using Preparation I, this decrease after 12 months of storage was 5.1% in bottles and 18.9% in the minims.

The viscosity of the drops prepared using Preparation II changed significantly after 6 months of their storage at 25 °C; i.e., 22.4% for drops stored in bottles and 29.7% for minims (Table 5). After analyzing the viscosity parameter for drops stored at 25 °C and 40 °C, we concluded that the drop in viscosity of the drops was less observed when stored in bottles than in minims. (Table 3 and Table 5). On the other hand, lowering the storage temperature to 2–8 °C had a stabilizing effect on this parameter. The observed drop in the viscosity of drops stored for 12 months in minims was only 2.1% (Table 5). Meanwhile, the following color changes of the drops were observed: for Preparation I, after 9 months of storage at 25 °C, and for Preparation II—after 3 months. The drops were stored in a refrigerator (2–8 °C) and showed no discoloration.

### 2.3. Chromatographic Purity of Eye Drops

**Figure 4:**
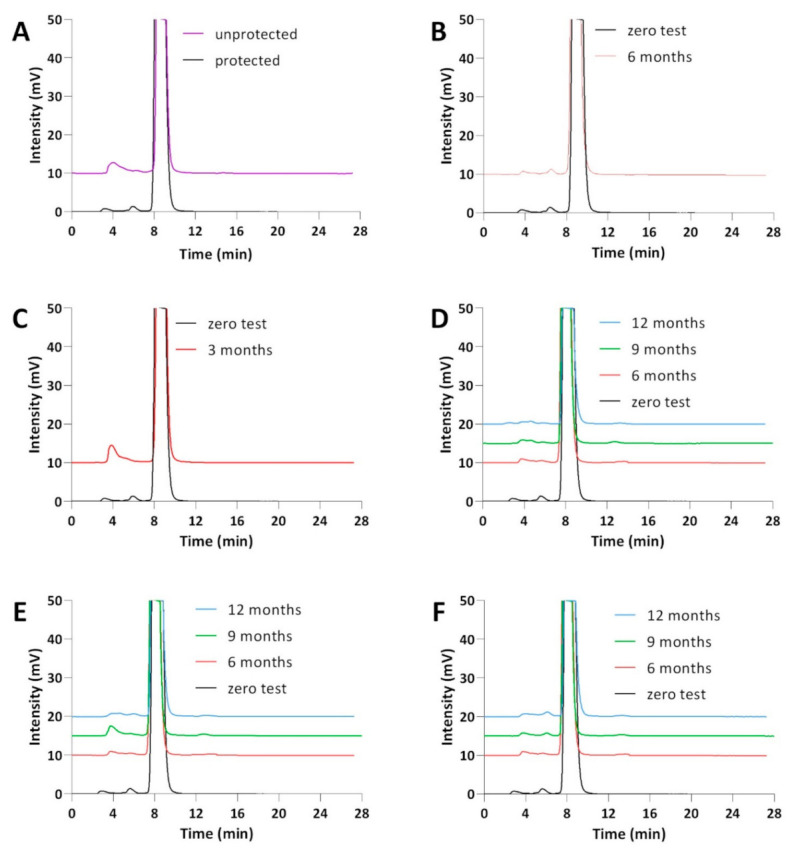
HPLC-UV chromatograms of eye drops with CS (2%) and HA (0.4%) from a chromatographic purity test: (**A**) after exposure to light (minims, 250 W/m^2^; 1.2 × 10^6^ lux·h); (**B**) stored in bottles under controlled conditions (40 °C/75% RH); (**C**) stored in minims under controlled conditions (40 °C/75% RH); (**D**) stored in bottles under controlled conditions (25 °C/60% RH); (**E**) stored in minims under controlled conditions (25 °C/60% RH); (**F**) stored in minims under uncontrolled conditions (2–8 °C).

The pharmacopeial method of salicylic acid purity analysis was used to assess the chemical purity of the eye drops [38]. Of the additional peaks (2.8–3.6 min—P1, and 5.8–6.4 min—P2) observed in the chromatograms of CS (2%) and HA (0.4%) (Figure 4), the P2 peak was identified in earlier studies as phenol—an impurity of the raw material [39] that cannot exceed a level of 0.001% in drops.

The assessment of the parameter of the sum of impurities on the chromatograms of freshly prepared drops and after 12 months of their storage under controlled (25 °C/60% RH) and uncontrolled (2–8 °C) conditions was 0.61% and 0.21–1.24%, respectively, both in bottles and minims (Table 6). The result (sum of impurities: 1.24%) observed in the drop chromatograms after 9 months of storage in minims at 25 °C was questionable. However, after 6 months of storage, for the drops under the accelerated test conditions (40 °C/75% RH) in bottles and minims, the sum of impurities was 2.33% and 2.88%, respectively (Table 6). The observed effect of the increase in CS concentration (Table 3) and the sum of additional peaks in the HPLC-UV chromatogram was a consequence of the leakage of the minims under elevated temperature conditions (Table 6), as evidenced by the lack of peaks of salicylic acid decomposition products in the HPLC-MS/MS chromatograms (Figure 3).

In the chromatograms of the eye drops subjected to photodegradation in minims (1.2 × 10^6^ lux·h), the sum of the observed impurities increased from 0.41% to 1.99% compared to the freshly prepared solution (Table 6). According to the HPLC-MS/MS analysis, product P1 was observed in these conditions, being a mixture of 2,3- and 2,5-dihydroxybenzoic acid (Figure 3).

### 2.4. The Impact of Tested Eye Drops on SIRC Cell Line Viability

**Figure 5:**
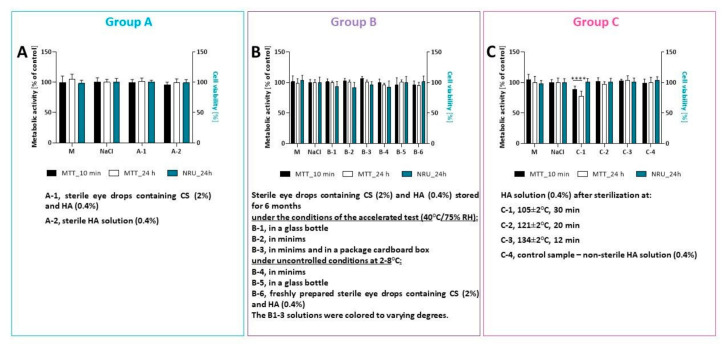
The effect of tested eye drops on SIRC cell viability: (**A**) cell viability after incubation with eye drops containing choline salicylate; (**B**) results for eye drops containing choline salicylate and hyaluronic acid after storage at different conditions; (**C**) data for eye drops containing hyaluronic acid and sterilized at different conditions. Sterile 0.9% NaCl was used as a control. Moreover, an additional control—cell culture medium (M) only—was also used. Cell viability was measured by MTT assay immediately after incubation with tested solutions and 24 h after incubation. Moreover, the cell viability was measured using the NRU assay (24 h after incubation with tested eye drops). Data are presented as a mean value + SD from two independent experiments. Statistical significance (****) between groups was assessed by Dunnett’s multiple comparison (*p* < 0.0001).

For many years, the SIRC cell line has served as an in vitro model to mimic corneal epithelium in pharmacological and toxicological studies [27,40,41]. To date, several assays have been validated and approved for assessing the ocular toxicity of ophthalmic drugs, such as neutral red uptake (NRU), fluorescein leakage (FL), or MTT assay [42]. We used the modified protocol for short-time incubation studies to determine the effect of tested solutions on SIRC cell viability [27,41,43,44,45]. In this study, only the undiluted solutions were used to mimic the situation immediately after application [44]. The 10 min exposure time represented the actual residence time of the applied solution on an ocular surface, and seemed to be the minimum period required for in vitro testing [44]. The cell viability was measured using the MTT and neutral red uptake assay immediately after 10 min of incubation and following a 24 h period (Figure 5). This recovery period could investigate the eventual reversibility of the cytotoxicity or delayed cytotoxic effect [45]. Short-time incubation confirmed that tested solutions A-1, A-2, B-(1–5), and C-(2–4) were well tolerated by the cells (cell viability > 90%), while the solution C-1 (HA solution after sterilization at 105 ± 2 °C, 30 min) exerted a statistically significant cytotoxic effect (88.54 ± 5.48% for MTT performed immediately after exposure and 77.52 ± 8.14% of cell viability after 24 h) (Figure 5).

Results from the NRU assay showed that all tested eye drops did not affect cell viability. Neutral red uptake used the ability of viable cells to incorporate and bind the dye neutral red in the lysosome [46]. The MTT assay measured the activity of a mitochondrial enzyme (succinate dehydrogenase), which converts MTT (a yellow substrate) into formazan (insoluble in water, purple product). This reaction occurs only in cells that are metabolically active (viable) [33]. These results suggest that solution C-1 may have affected mitochondria and the metabolic activity of SIRC cells. For the other solutions, the MTT and neutral red assays produced comparable results. Therefore, the sterilization process at 121 ± 2 °C (20 min) and the discoloration during eye drop storage did not affect their safety of use.

Microscopy revealed that the morphology of SIRC treated with tested solutions was similar to that of the control cells (Figures S1–S4).

### 2.5. Analysis of the Stability of HA Solutions and CS with HA Interactions by Size-Exclusion Chromatography

**Figure 6:**
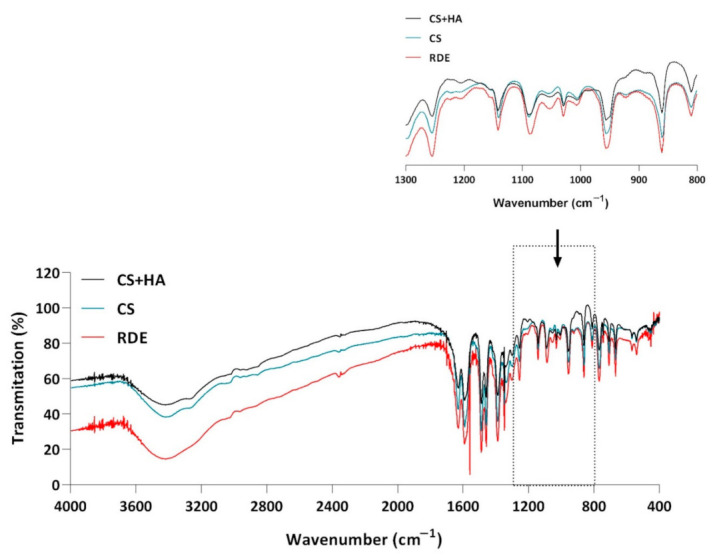
FTIR spectrum of choline salicylate (CS), a physical mixture of hyaluronic acid and choline salicylate (HA + CS), and residues after evaporation of eye drops with CS and HA (RDE) after 6 months of storage under accelerated test conditions (40 °C/75% RH).

Due to the results of the chemical purity analysis discussed above and the discoloration of the CS and HA eye drops observed during storage, we decided to perform a comparative analysis of the IR (FTIR) spectra of the physical mixture of CS and HA, CS, and the residues after the evaporation of the eye drops. However, no changes were observed in the IR spectrum, both in its entire range and in the so-called dactyloscopic area (Figure 6). Therefore, this study did not answer the question concerning the nature of the observed noncompliance.

**Figure 7:**
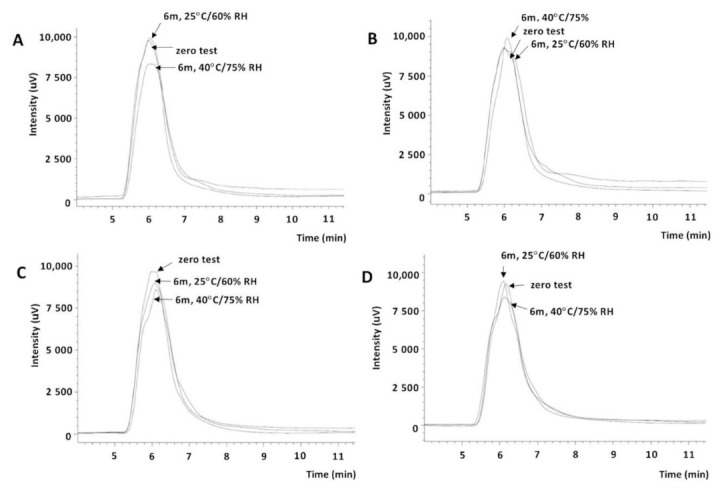
SE C-RID chromatograms: (**A**) HA (0.4%) solution without sterilization and after 6 months of controlled storage; (**B**) HA (0.4%) solution after sterilization at 105 °C (30 min) and after 6 months of controlled storage; (**C**) HA (0.4%) solution after sterilization at 121 °C (20 min) and after 6 months of controlled storage; (**D**) HA (0.4%) solution after sterilization at 134 °C (11 min) and after 6 months of controlled storage (25 °C/60% RH and 40 °C/75% RH).

Thus, in further study, size-exclusion chromatography was also used. Two types of detectors were used: UV (230 nm) and refractometric (RI). HA solutions were applied to the column (Yarra™ SEC-4000) before and after sterilization. The HA peak was only seen in the chromatograms recorded with RI detection. The influence of temperature and time of this process on the parameters of the HA peak was observed (Figure 7, Table 7).

Changes in the retention time, surface area, and asymmetry coefficient indicated the polymer degradation process. Szabó et al. observed a change in the molecular weight of HA during 20 min of sterilization at 100 °C from 1.4 MDa to 0.8 MDa, and suggested the sterilization of HA solutions by filtration, while HA was in powder form using radiation sterilization [31]. The analysis of the observed differences in the chromatograms showed that sterilization at 121 °C (20 min) slightly influenced the degradation of HA (0.36% compared to a nonsterile solution); such a solution was stored under long-term (25 °C/60% RH) and accelerated test conditions (40 °C/75% RH), and was sufficiently stable—the asymmetric ratio of the HA peak varied from −0.079 to −0.097 (Figure 7C, Table 7). The selected sterilization parameters (121 ± 2 °C, 20 min) could, therefore, be considered optional. Bothner et al. observed a decrease in the viscosity of the high-molecular-weight HA solutions after 10, 30, and 70 min of sterilization at 128 °C [28]. The viscosity tests of the prepared 0.4% HA solutions before and after sterilization with saturated steam under various conditions confirmed the decrease in viscosity depending on the temperature and time of sterilization and the conditions and storage time (Table 8). HA solutions sterilized at 121 °C (20 min) and stored for 6 months under the long-term test conditions showed a decrease in viscosity by approximately 31.2% and by approximately 39.6% in the accelerated test conditions. At the same time and under the same conditions, the viscosity of nonsterile HA solutions decreased by approximately 22.4% (Table 8). Thus, both HA solutions and eye drops (Table 5) should be stored under reduced temperature conditions (2–8 °C) to potentially limit this process.

**Figure 8:**
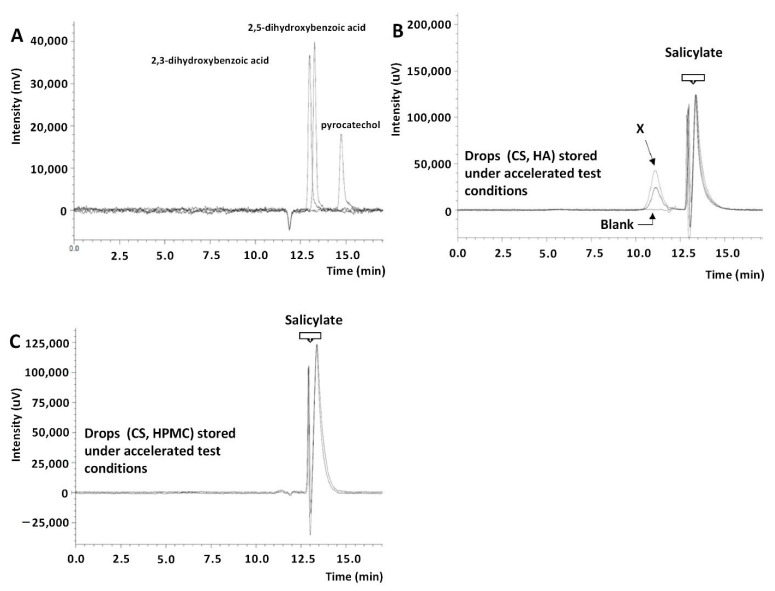
SEC-UV chromatograms: (**A**) standard solutions of CS photodegradation products; (**B**) eye drop solutions with CS (2%) and HA (0.4%) stored under accelerated test conditions (40 °C/75% RH) and a blank test solution; (**C**) eye drop solutions with CS (2%) and HPMC (0.5%) stored under accelerated test conditions (40 °C/75% RH).

At the same time, solutions of the reference substances were applied to the column: 2,3-, 2,5-dihydroxycarboxylic acid and pyrocatechin, colored as a result of storing CS (2%) and HA (0.4%) eye drops, and the blank test; i.e., HA-free drops. In the chromatograms of the colored eye drops (recorded with UV detection), apart from the salicylate peak (t_R_ approximately 13.5 min), an unidentified X peak with a retention time of 11.1 min (Figure 8B) was observed, which was not consistent with the 2,3- acid retention times, 2,5-dicarboxylic acid (13, 13.3 min), and pyrocatechin (14.7 min) (Figure 8A). For this reason, to confirm that this was an effect of CS–HA interaction, a CS and HPMC eye drop solution was applied to the column, for which no discoloration was observed during storage [26] or after storage for at least 3 months under accelerated test conditions. The X peak was not found in the chromatogram of this solution (Figure 8C).

## 3. Materials and Methods

### 3.1. Materials

The following substances and reagents were used in this research: choline salicylate (CS, 98.4%, ICN Polfa Rzeszow SA, Rzeszow, Poland); hyaluronic acid sodium salt P100 (1.52 MDa, CPN Sp. s ro, Dolni Dobrouc, Czech Republic; pyrocatechin (≥99.5%), 2,3- acid (99%), and 2,5-dihydroxybenzoic acid (98%) (Sigma-Aldrich, St. Louis, MO, USA); methanol for HPLC, propyl 4-hydroxybenzoate (99%, internal standard) (Avantor Performance Materials Poland SA, Gliwice, Poland); liquid thioglycolate substrate (TG; Cherwell Laboratories Ltd., Bicester, UK); Trypsin Soy Broth (TSB) according to EP + USP (Merck Sp. z o.o., Warsaw, Poland); and sodium chloride and peptone broth (pH 7.0, OXOID, UK), cleanliness class for analysis.

A cytotoxicity study was performed with a rabbit corneal cell line (SIRC) obtained from the European Collection of Cell Cultures (ECACC, Salisbury, UK). Unless otherwise stated, all cell culture reagents used in the experiments were purchased from Sigma-Aldrich (St. Louis, MO, USA). The cell line was grown in DMEM medium, without phenol red, supplemented with 10% (*v*/*v*) FBS; 1% (*v*/*v*), 10,000 penicillin units; 10 mg/mL streptomycin solution; and 1% (*v*/*v*), 1% L-glutamine (200 mM). Cells were grown at 37 °C in a humidified atmosphere containing 5% CO_2_.

Eprus^®^ minims sterile 1 mL made of polyethylene (LDPE) (Eprus Sp. z o.o., Bielsko-Biała, Poland) was used as the immediate packaging of the eye drops.

The permeability studies used were: regenerated cellulose membranes, MWCO 12,000–14,000 (Visking^®^, Serva Electrophoresis GmbH, Heidelberg, Germany); and corneas obtained from pig eyeballs (domestic pig) (local slaughterhouse).

### 3.2. Equipment and HPLC Conditions

#### 3.2.1. HPLC-UV

Liquid chromatography with UV detection (HPLC-UV) was used to determine CS in eye drops and to analyze the chemical purity of the drops. The device was a Shimadzu LC-10AT VP liquid chromatograph with SPD-10A VP UV–vis detector (Shimadzu, Kyoto, Japan), Rheodyne 7120 with 20 µL dosing loop; stationary phase: Nucleosil 100 C18, 4.6 mm × 150 mm, 5 µm (MZ Analytical); mobile phase: methanol-water-acetic acid (60:40:1, *v*/*v*/*v*), flow rate 1.0 mL/min for the assay and 0.5 mL/min for the purity analysis; detection at 230 nm or 270 nm for determination and purity analysis, respectively [39]. Detailed validation information was published in our previous paper [39].

#### 3.2.2. SEC-UV/RID

An HPLC Prominence-i LC-2030C Plus system with UV–vis and a RID-20A refractometric detector operated with LabSolutions DB—GPC software v.6.87 (all from Shimadzu, Kyoto, Japan) were used for stability analysis of HA solutions and prepared eye drops. Aqueous gel filtration chromatography (GFC) with UV at 230 nm [47] and RI detection (RID) was performed in a Yarra^TM^ SEC-4000 column (300 mm × 7.8 mm, 3 µm ultra-pure silica particle size, 500 Å pore size) with a SecurityGuard^TM^ Cartridge GFC-4000 (4 mm × 3.0 mm) System (all from Phenomenex^®^, Torrance, CA, USA), at 30 °C. The running buffer was a mixture of 0.2 M NaNO_3_ and 0.01 M NaH_2_PO_4_ (pH 7.0) at a flow rate of 1.0 mL/min [48]. The mobile phase was prefiltered through an OlimPeak^TM^ 0.2 μm hydrophilic PTFE filter (Teknokroma, Barcelona, Spain) and degassed on an ultrasonic degasser. Injections of 20 µL were made. The temperature of both detectors was 40 °C.

#### 3.2.3. HPLC-MS/MS

The HPLC-MS/MS assays were performed using a 1260 Infinity liquid chromatograph (Agilent Technologies, Santa Clara, CA, USA) with a triple quadrupole tandem mass spectrometer 4000 QTRAP (Sciex, Framingham, MA, USA). A TurboV ion source operating in negative ion mode (electrospray ionization—ESI) was used. Chromatographic separation was accomplished by applying a Nucleosil 100 C18 column, 4.6 × 150 mm, 5 µm (MZ Analytical) at 20 °C. Isocratic elution was used with a mobile phase consisting of 1% formic acid in a methanol–water mixture 60:40 (*v*/*v*) and a flow rate of 1000 µL/min. The injection volume was set at 5.00 µL. The total run time was 10 min.

An 11 PLUS syringe pump (Harvard Apparatus, Holliston, MA, USA) coupled directly to the ESI ion source of the mass spectrometer was used to select and optimize the ion transitions. These procedures were conducted by injecting methanolic–water solutions at 1:1 (*v*/*v*) of compounds of interest to the mass spectrometer at a flow rate of 10 µL/min. Final optimized conditions were as follows: ion spray voltage (IS): −4500.0 V; curtain gas nitrogen (CUR): 40.0 psi; temperature (TEM): 600 °C; nitrogen as ion source gas (GS1, GS2): 60.0 psi and 60.0 psi. Detection was performed on a mass spectrometer operated in multiple reaction monitoring (MRM) mode. Dwell time was set at 50.0 ms. The HPLC-MS/MS system was controlled, and the data acquisition and processing were conducted using Analyst Software version 1.6.3 (Sciex, Framingham, MA, USA) [39]. More validation information was published in our previous paper [39].

#### 3.2.4. Other Equipment

The spectrophotometric analysis was performed using a UV–vis Lambda 20 spectrophotometer (Perkin Elmer, Buckinghamshire, UK) and the FTIR IRAffinity-1 spectrophotometer (Shimadzu, Kyoto, Japan).

The following were used in the permeation studies: a Franz V6A-02 diffusion cell (Permegear, Inc., Hellertown, PA, USA), an Epsilon 2-4 LSC plus a freeze dryer (Martin Christ Gefriertrocknungsanlagen GmbH, Osterode am Harz, Germany), and a Bio RS-24 mini rotator (Biosan SIA, Riga, Latvia).

A Binder KBF 240 climatic chamber (Binder GmbH, Tuttlingen, Germany) with a Hydrolab Basic 10 water treatment system (Hydrolab Sp. z o.o., Sp. K., Straszyn, Poland) and Atlas Suntest CPS + (Atlas, Mount Prospect, IL, USA) were used for stability studies.

The physicochemical properties of the eye drops were assessed using a Shott Lab CG 842 pH meter (Schott Group, Mainz, Germany), a Marcel Os 3000 osmometer (Merazet SA, Poznan, Poland), and a HAAKE RheoStress1 rotational rheometer (ThermoScientific, Waltham, MA, USA) with a Thermo HAAKE DC 30 temperature controller.

A small steam sterilizer (Extacta, M.O. Com S.R.L., Montecchio, Emilia, Italy) was used for sterilization.

SIRC morphology was observed under a Nikon Eclipse TS100 microscope with an attached model C-SHG fluorescent unit and a DS-SMc digital camera. Absorbance was read using a Biotek Instruments plate reader, model Elx-800 (Highland Park, Winooski, VT, USA).

### 3.3. Methods

#### 3.3.1. Determination of CS in Eye Drops Using UV and HPLC-UV Methods

UV method: 0.2 mL of the eye drops was supplemented with water (or PBS in the permeation study) to 100.0 mL. The CS content was calculated using the parameters of the standard curve: y = (1.34 ± 0.01) 10^−2^ x [39] or y = (1.38 ± 0.01) 10^−2^ x for PBS buffer as solvent (x—CS concentration in µg/mL; y—absorbance at 296 nm). The parameters of the CS standard curve in PBS solution were determined in the range of 5.5–88.6 µg/mL [26]. The validation and revalidation information can be found in our previous papers [26,39].

HPLC-UV method: 0.1 mL of the test drops was added to 2.5 mL of methanolic propyl 4-hydroxybenzoate (1 mg/mL, i.s.) and made up to 25.0 mL with the mobile phase. The test and reference solutions (CS 2%) were applied to the chromatography column. The CS content in the drops was calculated as the arithmetic mean of three determinations.

#### 3.3.2. Chemical Purity by HPLC-UV and HPLC-MS/MS

HPLC-UV method: a solution of freshly prepared drops corresponding to a CS contamination of 0.1% (P0.1%) and a mixture of eye drops and a mobile phase (1:3, *v*/*v*) were applied to the column and developed for 30 min (0.5 mL/min, UV 270 nm).

HPLC-MS/MS method: a mixture of eye drops and a mobile phase (1:4, *v*/*v*) was applied to the column, and the presence of ions with retention times 2.47, 2.21, and 2.15 min (acid 2.3- and 2,5-dihydroxycarboxylic acid (152.9 → 108.9 and 152.9 → 80.9), pyrocatechin (108.9 → 90.9 and 108.9 → 80.9) [26].

#### 3.3.3. Analysis of Eye Drops’ pH, Osmolarity, and Viscosity

Each measurement was repeated three times and performed directly in the tested drops. The rheological tests were performed at a temperature of 25.0 ± 0.5 °C (geometry of the bob and Z20 cup; diameter: 20 mm, gap size: 4.2 mm).

#### 3.3.4. Sterility Test of Eye Drops

The sterility of the eye drops was tested following the requirements of the *European Pharmacopoeia* 10th Edition (2019; chapter 2.6.1. Sterility) [49] using the membrane filtration technique and direct inoculation of the culture medium [26]. Under the requirements of the *European Pharmacopoeia*, the suitability of the media and methods used was confirmed prior to the sterility test.

Evaluation of microbial growth was performed at one- or two-day intervals during 14-day incubation (34 ± 1 °C—TG and 22 ± 1 °C—TSB).

#### 3.3.5. Procedure for the Preparation of CS and HA Eye Drops

HA solution (0.8%): 0.8 g of HA was added to 99.2 g of water at room temperature and stirred for 3 h until completely dissolved. The resulting solution was allowed to swell for 24 h. The solution was then filtered through a Scott G-1 clarifier.

Eye drops with CS (2%) and HA (0.4%): 0.4 g of sodium chloride and 0.02 g of sodium bicarbonate were dissolved in 40.0 g of water, then 2.0 g of CS was added, mixed, and made up to 50.0 g with water (solution A). Then 50.0 g of HA solution (0.8%) was added to solution A, mixed, transferred to glass bottles, sealed, and autoclaved at 121 ± 2 °C for 20 min (Preparation I). In another procedure, 50.0 g of solution A was filtered through a sterilization filter (0.22 µm) into a sterile bottle, 50.0 g of a sterile 0.8% HA solution was added, and the bottle was closed with a sterile stopper under sterile conditions (Preparation II). The eye drops (Preparations I and II) were then aseptically transferred to the immediate packaging (Eprus^®^, 1 mL).

HA solution (0.4%) for stability and interaction studies: 0.4 g of sodium chloride was added and dissolved in 49.6 g of water at room temperature. Then, 50.0 g of a 0.8% HA solution was added, mixed, and filtered through a Scott G-1 caliper filter. The solution was divided into three parts, transferred to glass bottles, capped, and autoclaved at 121 ± 2 °C for 20 min, 134 ± 2 °C for 12 min, and 105 ± 2 °C for 30 min, respectively.

#### 3.3.6. Penetration through a Hydrophilic Membrane

Phosphate buffer pH 7.4 (PBS): 8.0 g of sodium chloride, 1.44 g of disodium hydrogen phosphate, 0.24 g of potassium dihydrogen phosphate, and 0.2 g of potassium chloride were weighed and dissolved in 900 mL of distilled water. The solution was then adjusted to pH 7.4 (HCl or NaOH 0.2 M) and made up to 1000.0 mL with water.

Test for CS permeation through a hydrophilic membrane: the test was performed at 35 ± 0.5 °C and 200 rpm with 8.0 mL of PBS buffer as acceptor. A membrane made of regenerated cellulose (Visking^®^) was presoaked in PBS solution (24 h, room temperature). CS eye drops (20 mg/mL) were placed in the donor chamber (1.0 mL), and at the appropriate time, 1.0 mL of the solution was withdrawn from the acceptor chamber and immediately replaced with fresh acceptor fluid. Then, the withdrawn solution (1.0 mL) was diluted to 25.0 mL with PBS buffer. The CS content was determined by the UV method by repeating the determination three times. At the same time, a study of the permeation of CS from its isotonic solution (2% CS in 0.99% NaCl) was performed. A blank test was performed to confirm the selectivity of the method.

#### 3.3.7. Penetration through a Porcine Cornea

Ex vivo porcine corneal CS permeation test: the procedure described in Section 2.5 was followed by placing a preaerated PBS buffer solution into the acceptor part of the Franz cell. Two time frames (5 min and 3 h) were adopted, after which the acceptor solution was withdrawn and diluted to 10.0 mL with PBS buffer. In addition, 0.2 mL of the solution was withdrawn from the donor portion in parallel and diluted to 100.0 mL with water. The CS content in the acceptor and donor parts was determined by the UV method. The examination was performed twice for each of the analyses described (after 5 min and 3 h), each time using a new cornea. Each sample was analyzed in triplicate. A blank test was performed to confirm the selectivity of the method.

After the permeation test, the corneas were freeze-dried (21 h, −35 °C, 0.2 mbar) after washing 3 times with distilled water.

Extraction of CS from the lyophilized cornea: extractions were carried out 3 times with a mixture (7.0 mL) of water–methanol (1:2, *v*/*v*) at room temperature (minirotor). The supernatant solutions were pooled, diluted to 25.0 mL with the same solvent, and filtered (0.2 µm). Further, 3.0 mL of the filtrate was made up to 10.0 mL with the same solvent. The content of CS was determined (UV method) using a solution of CS in a mixture of water and methanol (1:2) (60.5 µg/mL) as a reference solution.

#### 3.3.8. Eye Drop Stability

The eye drops were photodegraded in direct minims packaging in an Atlas Suntest CPS + device (250 W/m^2^; 1.2 × 10^6^ lux·h). At the same time, the drops were exposed to light in the immediate packaging, protecting them from light with aluminum foil. HPLC-UV and HPLC-MS/MS were used to analyze the degree of CS photodegradation and the presence of its products.

The influence of temperature and air humidity on the stability of drops stored in bottles and minims was carried out under accelerated test conditions (40°/75% RH; RH—relative humidity), in a long-term test (25 °C/60% RH), and in uncontrolled conditions (2–8 °C). The HPLC-UV method determined the CS content and the drop quality (pH, osmolarity, viscosity), and HPLC purity and microbiological purity were assessed.

#### 3.3.9. Cytotoxicity of Tested Eye Drops Using the Modified STE Assay

The following solutions were used to test the cytotoxic effect on the SIRC cell line:

Group A—sterile preparation (2% CS and 0.4% HA: A-1); control sample—sterile 0.4% HA solution (A-2).

Group B—sterile preparation (2% CS and 0.4% HA) stored for 6 months (40 °C/75% RH): in a glass bottle (B-1), in minims (B-2), and in minims and a package cardboard box (B-3); in uncontrolled conditions at 2–8 °C: in minims (B-4) and in a glass bottle (B-5); control sample—freshly prepared sterile eye drops containing CS (2%) and HA (0.4%) (B-6). The B1–3 solutions were colored to varying degrees.

Group C—HA solution (0.4%) after sterilization at: 105 ± 2 °C, 30 min (C-1); 121 ± 2 °C, 20 min (C-2); 134 ± 2 °C, 12 min (C-3); and control sample—nonsterile HA solution (0.4%) (C-4).

Sterile 0.9% NaCl was used as a control. Moreover, an additional control (cell culture medium only—M) was also used.

**Figure 9:**
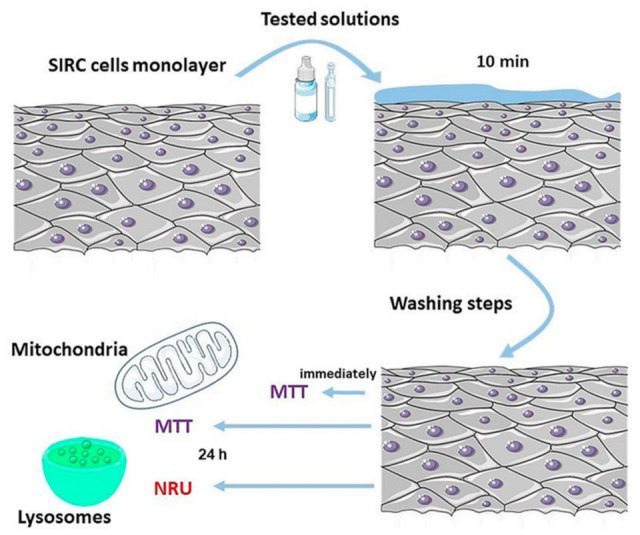
A schematic illustration of the in vitro experiments using the SIRC cell line. The impact of tested eye drops on cell viability was determined using MTT and NRU assays.

The schematic illustration of experimental protocols is presented in Figure 9.

MTT assay: Cells were seeded in 96-well plates at a density of 2 × 10^4^ cells per well and incubated for 48 h in cell culture conditions before starting an experiment. Subsequently, tested solutions and controls were added to wells, and cells were incubated for 10 min at room temperature. After incubation, the cells were rinsed twice with PBS. MTT assay was performed according to a previous report [27]. To test the cytotoxic effect immediately after incubation with the tested solution, for one plate, 170 µL of reaction solution containing methylthiazolydiphenyl-tetrazolium bromide (MTT) solution (5 mg/mL in PBS) in culture medium (final concentration 0.59 mg/mL) was added to each well. The plates were incubated for 2 h at 37 °C and then centrifuged at 1200 rpm for 3 min. The formazan crystals were extracted with 200 µL dimethylsulfoxide (DMSO). The absorbance was measured at 570 nm with a plate reader (Biotek Instruments, Elx-800). Cell viability was calculated as a percentage of the control.

To test the cytotoxic effect after 24 h, after incubation with the tested solutions (10 min), 150 µL of fresh medium was added to each well, and cells were incubated for a 24 h period. Then, the MTT assay, according to the described procedure, was performed.

Neutral red uptake (NRU) assay: SIRC cells were seeded at a density of 2 × 10^4^ cells per well in 96-well plates and incubated for 48 h under cell culture conditions. After incubation, the cells were exposed to the tested solutions for 10 min. Then, cells were washed twice with PBS, and 150 µL of fresh in the medium was added to each well. The cells were incubated for 24 h, and the NRU test was performed according to procedures previously described [40,50] with some modifications. Briefly, NRU solution (50 µg/mL in medium without FBS) was added to each well and incubated for 3 h at 37 °C. The dye was extracted with a mixture of 1% (*v*/*v*) acetic acid, 50% (*v*/*v*) ethanol, and 49% (*v*/*v*) sterile water, and the absorbance was read using a spectrophotometer at 570 nm with a plate reader (Biotek Instruments, Elx-800). The results were calculated as a percentage of the control.

Statistical analysis: The results are presented as the mean + SD from two independent experiments. The values were calculated using GraphPad Prism version 8.00 for Windows, GraphPad Software, San Diego, CA, USA. Data were compared for statistical significance by the Dunnett’s multiple comparison test; a probability value (*p*) of less than 0.05 was considered significant.

#### 3.3.10. Analysis of the Stability of HA Solutions and CS and HA Interactions by Size-Exclusion Chromatography

The following solutions were applied to the Yarra^TM^ SEC-4000 chromatographic column: hyaluronic acid (0.4%), nonsterile and sterilized at 105 ± 2 °C for 30 min, 121 ± 2 °C for 20 min, and 134 ± 2 °C for 12 min, as well as these solutions stored 6 months under accelerated test conditions (40 °C/75% RH); colored eye drop solutions containing CS (2%) and HA (0.4%) and eye drop solutions containing CS (2%) and HPMC (0.5%) after at least 3 months of storage under accelerated test conditions; freshly made eye drops with CS (2%) and HA (0.4%) (sterile and nonsterile) and stored for 12 months in uncontrolled conditions at 2–8 °C; blank test—CS drops (2%) without HA. Chromatograms were analyzed using UV (230 nm) and refractometric (RI) detection. To confirm or exclude the presence of photodegradation products of salicylic acid, solutions of the reference substances (0.1 mg/mL) of 2,3- and 2,5-dihydroxycarboxylic acid, and pyrocatechin in the mobile phase, were applied to the column. Each solution was injected in triplicate to the column and the retention times, surface areas, and asymmetry coefficients of the observed peaks were recorded.

#### 3.3.11. Analysis of CS and HA Interactions Using FTIR

KBr tablets were prepared, and the following FTIR spectra were made: CS, CS and HA equilibrium mixture, and the residue after evaporation of stained eye drops stored for a minimum of 3 months (40 °C/75% RH).

## 4. Conclusions

The presented methodology can be applied in the hospital or community pharmacy to prepare eye drops containing CS and HA under aseptic conditions. The developed formula of eye drops with CS (2%) and HA (0.4%) met the criteria of stability and safety when the product was stored in conditions of no light access and at a temperature of 2–8 °C. The maximum permissible level of the sum of the pollutants in this case was taken as 1%. The color changes observed during storage at higher temperatures indicated the potential interaction in the pharmaceutical phase. Notably, this effect was observed only in drops that contained both compounds—CS and HA. Further research should clarify this phenomenon, since the interaction product of these two compounds was not observed immediately after sterilization of the drops. Whether the applied drop sterilization technique was the factor initiating the process is yet to be determined. Did the change in the sterilization method of the HA solution (filtration technique—sterilizing filtration of the polymer solution is not possible in the clinical pharmacy practice; e.g., in a hospital pharmacy), or the finished product affect this process? Was there an interaction between CS and HA, or possibly between CS and HA degradation products? Despite the cytotoxicity results indicating that the vitality of the corneal cells was not affected after the application of these eye drops, their quality defect in the form of discoloration limits their use by determining their shelf life.

Considering the lack of a preservative in the prepared drops, minims can be used as a single-dose immediate packaging. Sterile Eprus^®^ minims (1 mL) met the requirements of maintaining the sterility of the drops and ensured the stability of the drops in conditions lacking access to light and at temperatures no higher than 2–8 °C. Eye drops with CS (2%) and HA (0.4%) stored in minims at 2–8 °C in the absence of light for 12 months showed no signs of discoloration.

The significance of the research was to design novel eye drops for treating corneal diseases. This study demonstrated the promising potential of eye drops based on hyaluronic acid to deliver choline salicylate efficiently by increasing the viscosity. Moreover, hyaluronic acid may itself serve as a therapeutic agent. After appropriate clinical trials, the developed formulation can be introduced as an eye preparation with a local anti-inflammatory, regenerating, and moisturizing effect.

## Figures and Tables

**Table 1:** Kinetic study for CS permeation from the eye drops (CS 20.2 mg/mL, HA 0.4%) through a hydrophilic membrane made of regenerated cellulose (Visking^®^).

Kinetic Model	Equation	Kinetic Parameter	-r
Zero-order	$\left(Q_{\infty }-Q_{t}\right)=Q_{\infty }-Q_{0})-k\;\cdot t$	*k* = (4.50 ± 0.53)·10^−2^, mg·min^−1^	0.9949
First-order	$ln\left(Q_{\infty }-Q_{t}\right)=ln\left(Q_{\infty }-Q_{0}\right)-k\;\cdot t$	*k* = (5.22 ± 1.09)·10^−3^, min^−1^	0.9840
Higuchi	$Q_{t}=k_{H}\;\cdot t^{1/2}$	*K_H_* = (9.42 ± 1.98)·10^−1^, min^−1/2^	0.9837
Korsmeyer–Peppas	$\frac{M_{t}}{M_{\infty }}=k_{r}\;\cdot t^{n}$	*k_r_* = (8.40 ± 0.43)·10^−3^, min^−n^, *n* = 0.80	0.9869

Note: *Q*_0_, *Q_t_*, *Q*_∞_—CS content at time *t*_0_, *t*, and *t*_∞_, respectively, in mg; *M_t_*/*M*_∞_—CS fraction at time *t* and *t*_∞_, respectively; *t*—time, in min; *k*, *K_H_*, and *k_r_*—permeation rate constants.

**Table 2:** The amount of CS determined in the donor part of the Franz cell and in the porcine cornea after 5 min and 3 h corneal exposure to eye drops (CS 20.4 mg/mL, HA 0.4%) (mean ± SD, *n* = 3).

Solution	Time	Donor Chamber		Porcine Cornea	
		mg	%	mg	%
CS (20.4 mg/mL) with HA (0.4%)	5 min	17.29 ± 0.25	84.8 ± 1.2	1.60 ± 0.04	7.8 ± 0.2
	3 h	16.67 ± 0.01	81.7 ± 0.1	3.74 ± 0.05	18.3 ± 0.2

**Table 3:** Stability of choline salicylate eye drops formulated using Preparations I and II stored under controlled conditions (40 °C/75% RH) in bottles and minims—results of CS determinations by HPLC and measurement of pH, viscosity, and osmolarity (mean ± SD, *n* = 3).

Package	Time, Months	Determined Concentration, %	Percentage of Declared Value, %	pH	Viscosity, mPa·s	Osmolarity, mOsm/L
**Preparation I**						
Bottle	0	2.12 ± 0.09	100.0 ± 4.2	7.41 ± 0.05	9.56 ± 0.06	314 ± 2
	1	2.09 ± 0.08	98.6 ± 3.8	7.41 ± 0.03	Nd	312 ± 1
	2	2.07 ± 0.05	98.5 ± 2.4	7.41 ± 0.03	Nd	313 ± 1
	3	2.10 ± 0.06	96.5 ± 2.8	7.40 ± 0.01	9.38 ± 0.04	311 ± 1
	4	2.00 ± 0.04	96.1 ± 1.9	7.41 ± 0.07	Nd	312 ± 1
	5	2.00 ± 0.08	96.5 ± 3.8	7.40 ± 0.01	Nd	311 ± 2
	6	2.14 ± 0.11	98.5 ± 5.2	7.41 ± 0.08	9.24 ± 0.05	312 ± 2
Minims	0	2.12 ± 0.09	100.0 ± 4.2	7.41 ± 0.05	9.56 ± 0.06	314 ± 2
	1	2.13 ± 0.04	100.5 ± 1.9	7.52 ± 0.02	Nd	313 ± 2
	2	2.18 ± 0.05	102.8 ± 2.4	7.63 ± 0.05	Nd	313 ± 2
	3	2.34 ± 0.09	110.4 ± 4.2	7.67 ± 0.03	7.45 ± 0.02	309 ± 2
**Preparation II**						
Bottle	0	2.01 ± 0.04	100.0 ± 2.0	7.80 ± 0.05	9.93 ± 0.02	298 ± 1
	6	2.01 ± 0.04	100.0 ± 2.0	7.61 ± 0.05	6.91 ± 0.06	320 ± 1
Minims	0	2.01 ± 0.04	100.0 ± 2.0	7.80 ± 0.05	9.93 ± 0.01	298 ± 1
	3	2.04 ± 0.06	101.5 ± 3.0	7.65 ± 0.03	Nd	300 ± 2
	6	2.32 ± 0.01	115.4 ± 0.5	7.50 ± 0.05	6.41 ± 0.05	298 ± 1

Note: Nd—no data.

**Table 4:** Microbiological purity test results for eye drops with CS (2%) and HA (0.4%) stored in immediate packaging (minims) under controlled conditions (40 °C/75% RH).

Time, Months	Sample	Sample Volume, mL	Direct Inoculation		Membrane Filtration	
			TSB	TG	TSB	TG
0	1	Whole content—1 mL	No growth	No growth	No growth	No growth
	2	Whole content—1 mL	No growth	No growth	No growth	No growth
	3	Half content—0.5 mL	No growth	No growth	No growth	No growth
3	1	Whole content—1 mL	Growth	No growth	No growth	No growth
	2	Whole content –1 mL	No growth	No growth	No growth	No growth
	3	Half content—0.5 mL	No growth	No growth	No growth	No growth

Note: TSB—tryptic soy broth; TG—fluid thioglycolate medium.

**Table 5:** Stability of choline salicylate eye drops formulated using Preparations I and II stored under controlled (25 °C/60% RH) and uncontrolled conditions (2–8 °C) in bottles and minims—results of CS determinations by HPLC and measurement of pH, viscosity, and osmolarity (mean ± SD, *n* = 3).

Package	Time, Months	Determined Concentration, %	Percentage of Declared Value, %	pH	Viscosity, mPa·s	Osmolarity, mOsm/L
Preparation I—controlled conditions (25 °C/60% RH)						
Bottle	0	2.00 ± 0.07	100.0 ± 3.5	7.41 ± 0.05	9.56 ± 0.06	314 ± 2
	3	2.01 ± 0.01	100.5 ± 0.5	7.39 ± 0.03	Nd	314 ± 1
	6	1.96 ± 0.03	98.0 ± 1.5	7.21 ± 0.02	9.35 ± 0.03	308 ± 2
	9	1.97 ± 0.01	98.5 ± 0.5	7.33 ± 0.05	Nd	310 ± 3
	12	2.03 ± 0.01	101.5 ± 0.5	6.99 ± 0.05	9.07 ± 0.07	311 ± 1
Minims	0	2.00 ± 0.07	100.0 ± 3.5	7.41 ± 0.05	9.56 ± 0.06	314 ± 2
	3	2.04 ± 0.01	102.0 ± 0.5	7.35 ± 0.04	Nd	310 ± 1
	6	2.07 ± 0.01	102.5 ± 0.5	7.29 ± 0.02	8.52 ± 0.05	309 ± 1
	9	2.02 ± 0.04	101.0 ± 2.0	7.18 ± 0.03	Nd	297 ± 3
	12	2.07 ± 0.12	103.5 ± 6.0	7.20 ± 0.05	7.75 ± 0.08	311 ± 2
Preparation I—uncontrolled conditions (2–8 °C)						
Minims	0	2.00 ± 0.07	100.0 ± 3.5	7.41 ± 0.05	9.56 ± 0.01	314 ± 2
	3	2.07 ± 0.02	103.5 ± 1.0	7.44 ± 0.05	Nd	312 ± 1
	6	2.08 ± 0.02	104.0 ± 1.0	7.33 ± 0.03	9.43 ± 0.04	305 ± 2
	9	2.07 ± 0.02	103.5 ± 1.0	7.39 ± 0.04	Nd	311 ± 1
	12	2.02 ± 0.01	101.0 ± 0.5	7.28 ± 0.02	9.36 ± 0.07	309 ± 2
Preparation II—controlled conditions (25 °C/60% RH)						
Bottle	0	2.01 ± 0.04	100.0 ± 2.0	7.80 ± 0.05	9.93 ± 0.01	298 ± 1
	6	2.10 ± 0.02	104.5 ± 1.0	7.40 ± 0.05	7.71 ± 0.01	291 ± 1
Minims	0	2.01 ± 0.04	100.0 ± 2.0	7.80 ± 0.05	9.93 ± 0.04	298 ± 1
	3	2.02 ± 0.02	100.5 ± 1.0	7.65 ± 0.02	7.70 ± 0.03	295 ± 2
	6	2.08 ± 0.02	103.5 ± 1.0	7.68 ± 0.03	6.98→ 0.06	290 ± 1

Note: Nd—no data.

**Table 6:** Chromatographic (HPLC-UV) purity analysis of CS (2%) with HA (0.4%) eye drops stored in bottles and minims under controlled and uncontrolled conditions, and after exposure to the light, showing the area (P) of the observed peaks (P_1_, P_2_, P_3_) and the percentage relative to the area of the main peak corresponding to the level of impurities of 0.1% (P_0.1%_ = 16862).

Storage Conditions	Package	Time, Months	Observed Peaks Outside the CS Main Peak				Sum of Impurities, %
			P_1_ t_R_ = 2.8–3.6 min		P_2_ t_R_ = 5.8–6.4 min		
			P	%	P	%	
40 °C/75% RH	Bottle	0	35650	0.21	69001	0.41	0.61
		6	31392	1.99	57993	0.34	2.33
	Minims	0	35650	0.21	69001	0.41	0.62
		3	486043	2.88	No		2.88
25 °C/60% RH	Bottle	0	35650	0.21	69001	0.41	0.61
		6	61142	0.36	48304	0.29	0.65
		9	35925	0.21	No		0.21
		12	28989	0.17	37973	0.22	0.39
	Minims	0	35650	0.21	69001	0.41	0.61
		6	73551	0.44	51080	0.30	0.74
		9	209457	1.24	No		1.24
		12	13879	0.08	No		0.08
2–8 °C	Minims	0	35650	0.21	69001	0.41	0.61
		6	43503	0.26	52962	0.31	0.57
		9	35936	0.21	50559	0.30	0.51
		12	11324	0.07	60556	0.36	0.44
Light exposure (250 W/m^2^; 1.2 × 10^6^ lux·h)							
	Package	Sample	P_1_ t_R_ = 2.8–4.2 min		P_2_ t_R_ = 5.8–6.4 min		Sum of impurities, %
			P	%	P	%	
	Minimis	protected	35590	0.21	33639	0.20	0.41
		unprotected	336527	1.99	No		1.99

Note: t_R_—retention time; P—area; P_1_, P_2_—impurities; No—not observed.

**Table 7:** Changes of parameters describing SEC-RID chromatograms (retention time, area, asymmetry factor) of HA (0.4%) solutions after sterilization and during storage under the conditions of the accelerated and long-term tests. Changes to the chromatograms of the unsterilized solution are given in brackets.

Sterilization Conditions	Fresh Solution			Observed Changes During Storage					
				25 °C/60%RH—6 Months			40 °C/75%RH—6 Months		
	t_R_, min	P	A_f_	t_R_, min	P	A_f_	t_R_, min	P	A_f_
Without sterilization	6.010	560078	1.765	+0.028	+4.71%	Nc	+0.016	−4.16%	+0.176
105 °C/30 min	5.998 (−0.012)	565425 (+0.95%)	1.848 (+0.083)	+0.003	+7.56%	+0.327	+0.081	−1.27%	+0.024
121 °C/20 min	5.997 (−0.013)	558036 (−0.36%)	1.793 (+0.028)	+0.071	+1.76%	−0.079	+0.115	−4.19%	−0.097
134 ^°C^/12 min	6.164 (+0.154)	546165 (−2.48%)	1.717 (−0.048)	−0.069	+1.29%	+0.034	−0.044	+1.33%	+0.215

Note: t_R_—retention time; P—area; A_f—_asymmetry factor; Nc—no change.

**Table 8:** Change in viscosity of 0.4% HA solutions in NaCl after sterilization and during storage under accelerated and long-term test conditions (mean ± SD, *n* = 3).

Sterilization Conditions	Fresh Solution Viscosity, mPa·s	Observed Changes During Storage	
		25 °C/60%RH—6 Months	40 °C/75%RH—6 Months
		Viscosity, mPa·s	Viscosity, mPa·s
Without sterilization	15.71 ± 0.01	12.19 ± 0.02	12.19 ± 0.02
105 °C/30 min	14.57 ± 0.01	11.36 ± 0.01	10.66 ± 0.01
121 °C/20 min	11.56 ± 0.01	7.95 ± 0.01	6.98 ± 0.02
134 °C/12 min	6.28 ± 0.01	5.56 ± 0.01	4.98 ± 0.01

## Data Availability

Data is contained within the article and supplementary material.

## Supplementary Materials

The following are available online at https://www.mdpi.com/article/10.3390/ph14090849/s1, Figure S1: Cell morphology 24 h after incubation with the tested solutions A-1, A-2, and C-(1–4), and appropriate controls; Figure S2: NRU staining 24 h after incubation with the tested solutions A-1, A-2, and C-(1–4), and appropriate controls; Figure S3: Cell morphology 24 h after incubation with the tested solutions B-(1–6), and appropriate controls; Figure S4: NRU staining 24 h after incubation with the tested solutions B-(1–6), and appropriate controls.
