# Supplementary material for: Novel Formulation of Eye Drops Containing Choline Salicylate and Hyaluronic Acid: Stability, Permeability, and Cytotoxicity Studies Using Alternative Ex Vivo and In Vitro Models
Source: Pharmaceuticals (Basel). 2021 Aug 26;14(9):849. doi: 10.3390/ph14090849 (PMC8465216; doi:10.3390/ph14090849)
Supplement: Supplementary file 1 [file pharmaceuticals-14-00849-s001.zip › pharmaceuticals-1321913-supplementary.pdf]

Novel Formulation of Eye Drops Containing Choline Salicylate and Hyaluronic Acid: Stability, Permeability, and Cytotoxicity Studies Using Alternative Ex Vivo and In Vitro Models

Katarzyna Barbara Wróblewska[1]*, Bartłomiej Milanowski [1,2], Małgorzata Kucińska [3]*, Szymon Plewa [4], Jolanta Długaszewska [5], and Izabela Muszalska-Kolos [6]

[1] Department of Pharmaceutical Technology, Poznan University of Medical Sciences, Grunwaldzka 6, 60-780 Poznan, Poland, bmilan@ump.edu.pl
[2] GENERICA Pharmaceutical Lab, Regionalne Centrum Zdrowia Sp. z o.o., Na Kępie 3, 64-360 Zbąszyń, Poland, b.milanowski@rcz-zbaszyn.pl
[3] Department of Toxicology, Poznan University of Medical Sciences, Dojazd 30, 60-631 Poznan, Poland,
[4] Department of Inorganic and Analytical Chemistry, Poznan University of Medical Sciences, Grunwaldzka 6, 60-780 Poznan, Poland, splewa@ump.edu.pl
[5] Department of Genetics and Pharmaceutical Microbiology, Poznan University of Medical Sciences, Święcickiego 6, 60-781 Poznan, Poland, jdlugasz@ump.edu.pl
[6] Department of Pharmaceutical Chemistry, Poznan University of Medical Sciences, Grunwaldzka 6, 60-780 Poznan, Poland, imuszals@ump.edu.pl

* Correspondence: kzajac@ump.edu.pl (K.B.W); kucinska@ump.edu.pl (M.K.)

Supplementary Data

Table of contents

Figure S1. Cells morphology 24h after incubation with the tested solutions A-1, A-2 and C-(1-4), and appropriate controls. 3

Figure S2. NRU staining 24h after incubation with the tested solutions A-1, A-2 and C-(1-4), and appropriate controls 4

Figure S3. Cells morphology 24h after incubation with the tested solutions B-(1-6), and appropriate controls. 5

Figure S4. NRU staining 24h after incubation with the tested solutions B-(1-6), and appropriate controls. 6

Abbreviations:

The following solutions were used to test the cytotoxic effect on the SIRC cell line:

Group A—sterile preparation (2% CS and 0.4% HA: A-1); control sample—sterile 0.4% HA solution (A-2).

Group B—sterile preparation (2% CS and 0.4% HA) stored for 6 months (40 °C/75% RH): in a glass bottle (B-1), in minims (B-2), and in minims and a package cardboard box (B-3); in uncontrolled conditions at 2–8 °C: in minims (B-4) and in a glass bottle (B-5); control sample—freshly prepared sterile eye drops containing CS (2%) and HA (0.4%) (B-6). The B1–3 solutions were colored to varying degrees.

Group C—HA solution (0.4%) after sterilization at: 105 ± 2 °C, 30 min (C-1); 121 ± 2 °C, 20 min (C-2); 134 ± 2 °C, 12 min (C-3); and control sample—nonsterile HA solution (0.4%) (C-4).

Sterile 0.9% NaCl was used as a control. Moreover, an additional control (cell culture medium only—M) was also used.

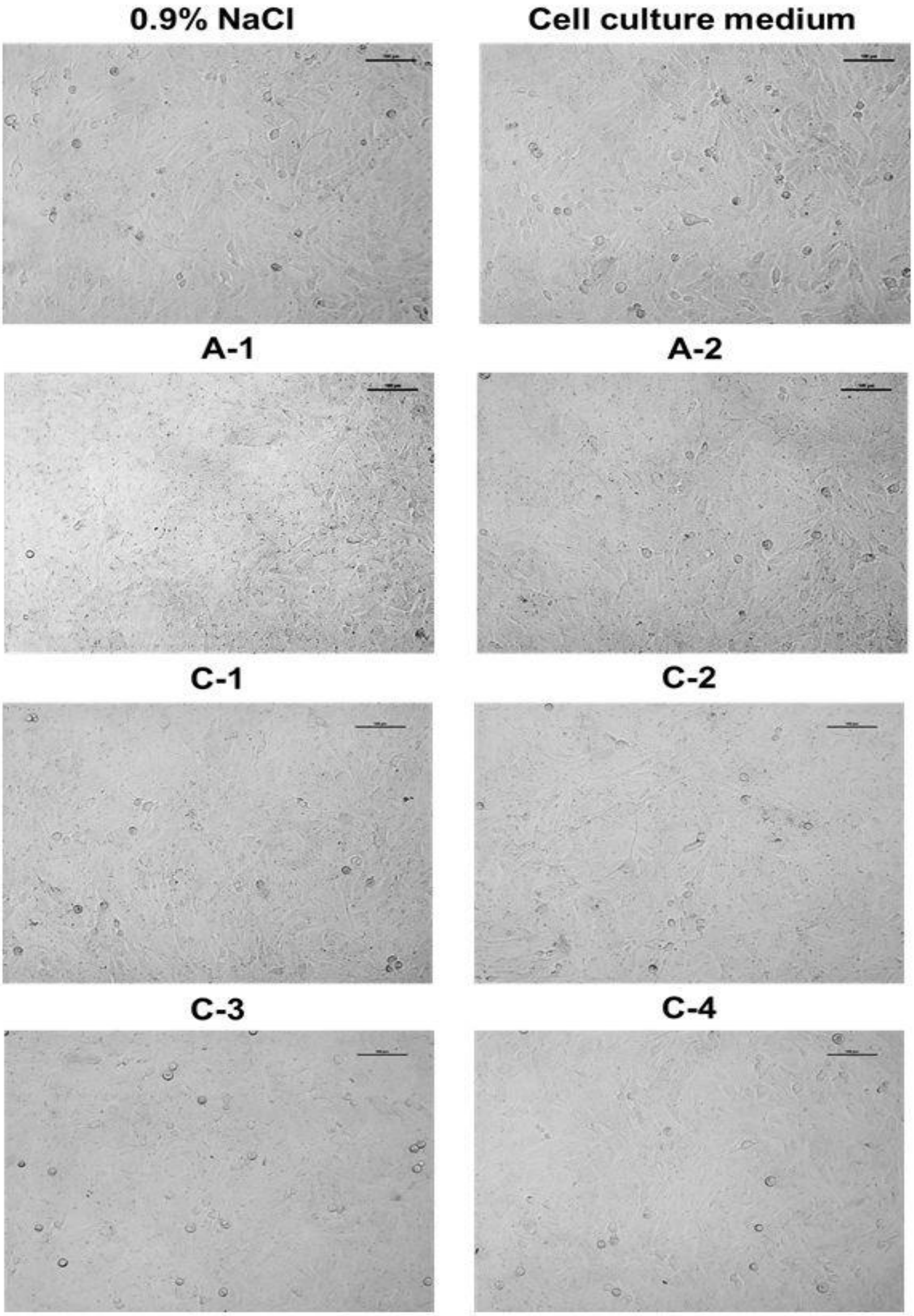

Figure S1. Cells morphology 24h after incubation with the tested solutions A-1, A-2 and C-(1-4), and appropriate controls. The images were taken with a DS-SMc digital camera attached to a Nikon Eclipse TS100 microscope. Scale bar corresponds to 100 μm.

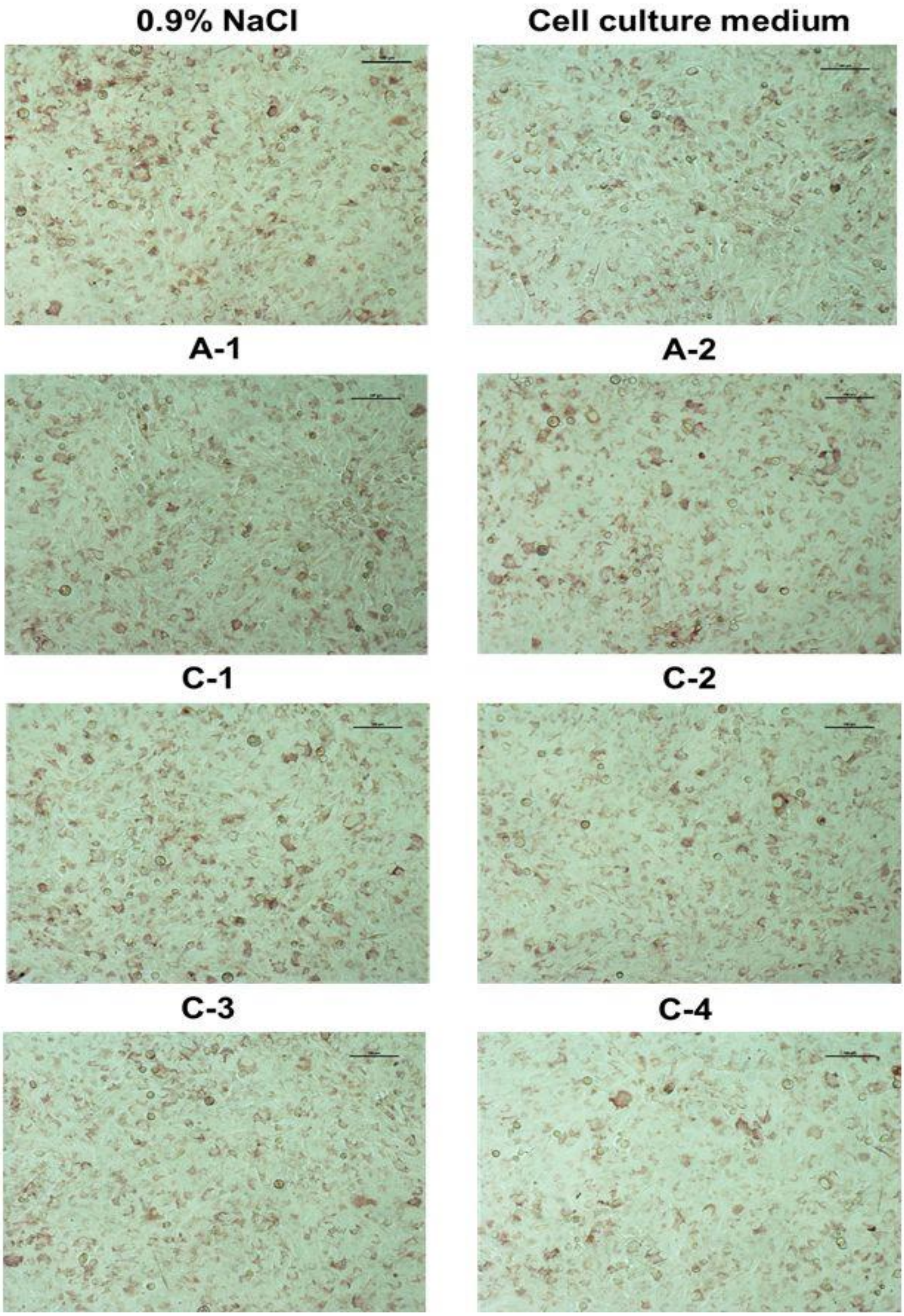

Figure S2. NRU staining 24h after incubation with the tested solutions A-1, A-2 and C-(1-4), and appropriate controls. The images were taken with a DS-SMc digital camera attached to a Nikon Eclipse TS100 microscope. Scale bar corresponds to 100 μm.

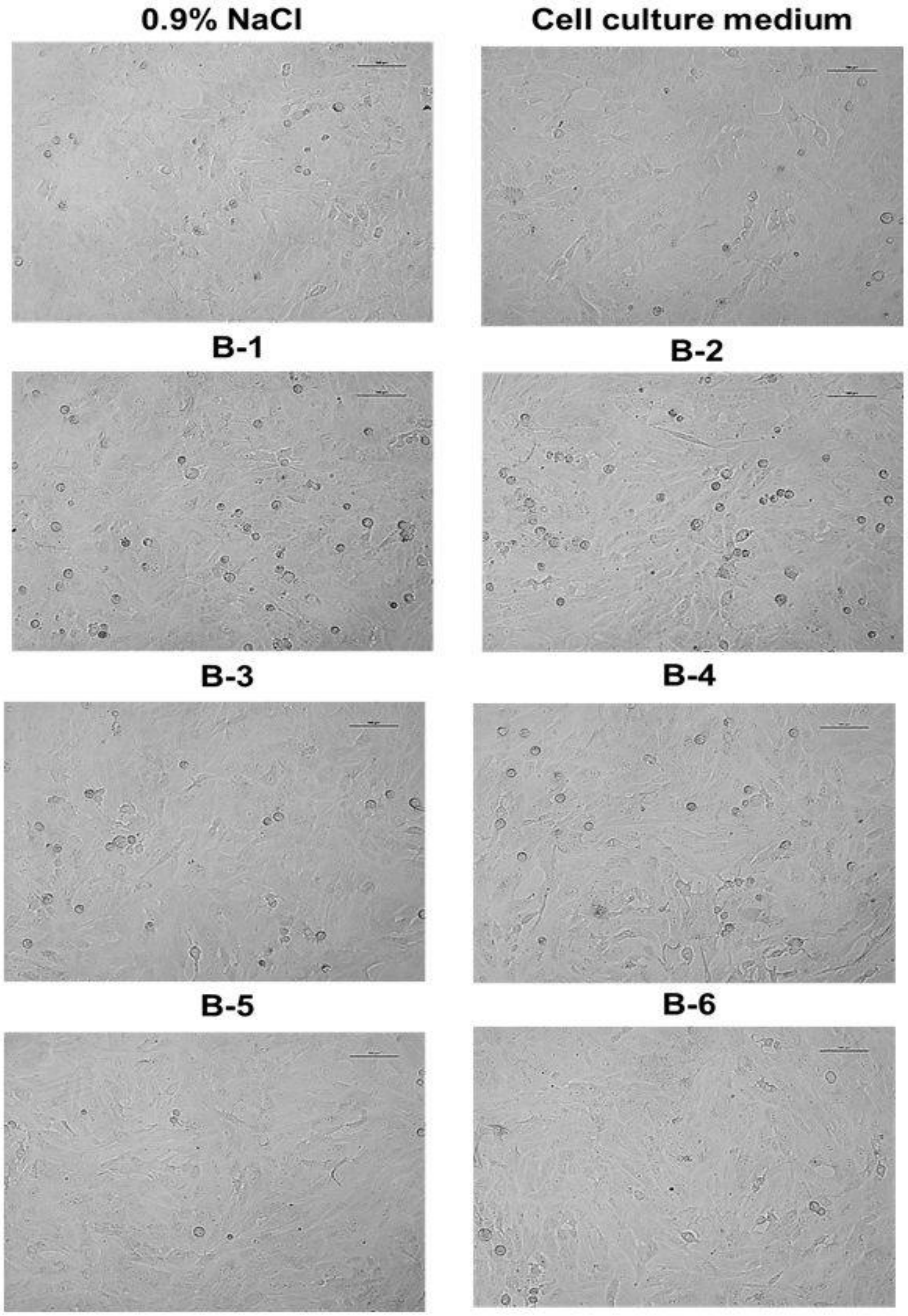

Figure S3. Cells morphology 24h after incubation with the tested solutions B-(1-6), and appropriate controls. The images were taken with a DS-SMc digital camera attached to a Nikon Eclipse TS100 microscope. Scale bar corresponds to 100 μm.

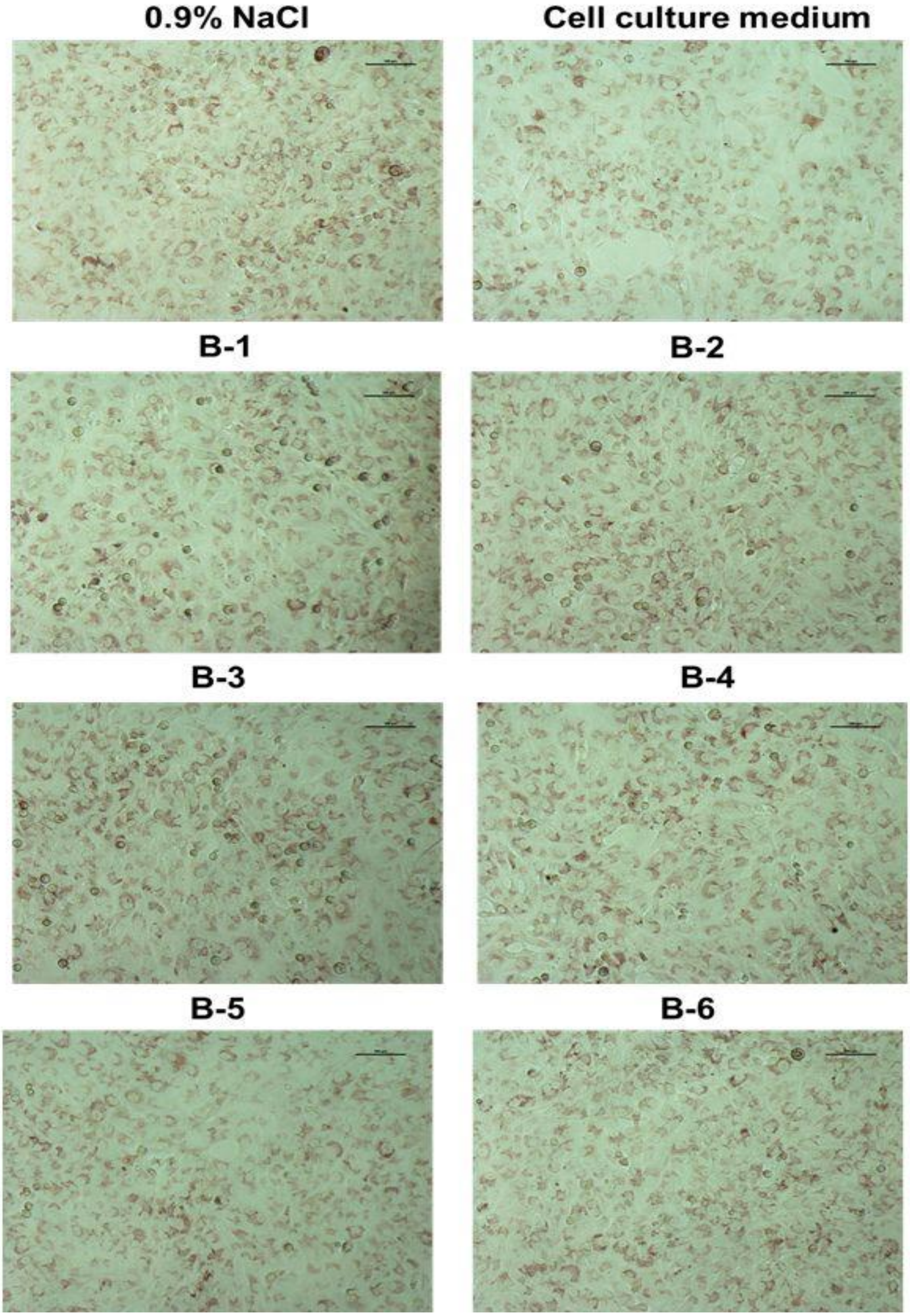

Figure S4. NRU staining 24h after incubation with the tested solutions B-(1-6), and appropriate controls. The images were taken with a DS-SMc digital camera attached to a Nikon Eclipse TS100 microscope. Scale bar corresponds to 100 μm.
